# HOIL-1L Interacting Protein (HOIP) as an NF-κB Regulating Component of the CD40 Signaling Complex

**DOI:** 10.1371/journal.pone.0011380

**Published:** 2010-06-30

**Authors:** Bruce S. Hostager, Daniel K. Fox, Douglas Whitten, Curtis G. Wilkerson, Betty A. Eipper, Victor P. Francone, Paul B. Rothman, John D. Colgan

**Affiliations:** 1 Department of Internal Medicine, University of Iowa, Iowa City, Iowa, United States of America; 2 Medical Scientist Training Program, University of Iowa, Iowa City, Iowa, United States of America; 3 Departments of Biochemistry and Molecular Biology and Plant Biology, Michigan State University, East Lansing, Michigan, United States of America; 4 Department of Neuroscience, University of Connecticut Health Center, Farmington, Connecticut, United States of America; 5 Department of Basic Sciences, The Commonwealth Medical College, Scranton, Pennsylvania, United States of America; New York University, United States of America

## Abstract

The tumor necrosis factor receptor (TNFR) superfamily mediates signals critical for regulation of the immune system. One family member, CD40, is important for the efficient activation of antibody-producing B cells and other antigen-presenting cells. The molecules and mechanisms that mediate CD40 signaling are only partially characterized. Proteins known to interact with the cytoplasmic domain of CD40 include members of the TNF receptor-associated factor (TRAF) family, which regulate signaling and serve as links to other signaling molecules. To identify additional proteins important for CD40 signaling, we used a combined stimulation/immunoprecipitation procedure to isolate CD40 signaling complexes from B cells and characterized the associated proteins by mass spectrometry. In addition to known CD40-interacting proteins, we detected SMAC/DIABLO, HTRA2/Omi, and HOIP/RNF31/PAUL/ZIBRA. We found that these previously unknown CD40-interacting partners were recruited in a TRAF2-dependent manner. HOIP is a ubiquitin ligase capable of mediating NF-κB activation through the ubiquitin-dependent activation of IKKγ. We found that a mutant HOIP molecule engineered to lack ubiquitin ligase activity inhibited the CD40-mediated activation of NF-κB. Together, our results demonstrate a powerful approach for the identification of signaling molecules associated with cell surface receptors and indicate an important role for the ubiquitin ligase activity of HOIP in proximal CD40 signaling.

## Introduction

Many members of the tumor necrosis factor receptor (TNFR) superfamily play critical roles in the regulation of immune responses. One member of this family, CD40, is a type I transmembrane protein expressed by antigen-presenting cells of the immune system, including macrophages, dendritic cells, and B cells [1], [2]. CD40 serves as a receptor for CD154, a cell surface protein expressed by activated T cells. The binding of CD154 to CD40 triggers signals in macrophages and dendritic cells that contribute to the activation of cell-mediated immune responses [1]. CD40 signals also promote humoral immune responses by helping to activate B cells to proliferate, differentiate, secrete antibody, and switch antibody isotypes [1], [2].

The mechanism by which CD40 transmits activation signals in antigen-presenting cells is only partially characterized. CD40, like many other members of the TNFR superfamily, interacts with intracellular proteins of the TNFR-associated factor (TRAF) family. These molecules link TNFR family members to downstream signaling molecules, such as NF-κB and stress-activated protein kinases [1]. Other proteins, including cIAP1 and subunits of the IKK enzyme complex, also appear to be recruited to CD40 [3]. To identify additional proteins participating in CD40 signaling, we used a combined stimulation/immunoprecipitation method to isolate CD40 signaling complexes from stimulated B cells. Analysis of the purified complexes by liquid chromatography/tandem mass spectrometry revealed the presence of many proteins known to associate with CD40, thus validating the approach. We used a CD40 mutant lacking the cytoplasmic tail to confirm the specificity of the interactions. We identified three novel CD40-associated proteins: SMAC, HTRA2, and HOIL-1L-interacting protein (HOIP). Western blot analysis of purified CD40 complexes confirmed our results. Recruitment of all three proteins was largely dependent on TRAF2, which has a critical role in CD40 signaling. Furthermore, we demonstrate that HOIP likely plays an important role in the CD40-mediated activation of NF-κB. Our results demonstrate a powerful method of isolating and identifying molecules associated with cell surface proteins and, more importantly, reveal previously unidentified and functionally significant components of the CD40 signaling apparatus.

## Results

### Isolation and identification of CD40-associated proteins

To identify novel components of the CD40 signaling complex, we employed a combined stimulation/immunoprecipitation protocol [4] designed to suit the physical properties of CD40 in activated cells. A somewhat similar approach has been used to isolate proteins associated with the T cell antigen receptor [5]. As previously shown, engagement of CD40 by its ligand or agonistic antibody results in the recruitment of the signaling complex to microdomains (membrane “rafts”) in the plasma membrane [6], [7]. Membrane microdomains tend to be insoluble in mild non-ionic detergents. Many immunoprecipitation protocols require the elimination of detergent-insoluble material from cell lysates prior to the addition of antibody-coated beads, and are therefore suboptimal for the isolation of CD40 signaling complexes. Although solubilization of microdomain-associated material is possible with stronger detergents, such treatment is likely to disrupt protein-protein interactions in the CD40 signaling complex. To avoid these difficulties, we used magnetic beads coated with anti-CD40 antibody to induce aggregation of CD40 and initiate signaling in live cells. After stimulation, the cells were disrupted with a mild detergent, leaving CD40 and its associated proteins on the beads. Beads were recovered by magnetic separation and then washed, thus allowing separation of the detergent-insoluble CD40 signaling complex from other detergent-insoluble material. We refer to this method as “activated receptor capture” (ARC) to indicate that the antibody-coated beads serve in cell stimulation as well as in the purification of the target molecule.

Although ARC purification proved to be an effective means of isolating signaling proteins associated with the cytoplasmic domain of CD40 (see below), the method does not preclude the co-purification of membrane-associated proteins irrelevant to CD40 signaling. For this reason, immunoprecipitation with a non-specific antibody does not serve as an appropriate negative control for ARC. In order to identify proteins specifically associated with the cytoplasmic domain of CD40, we compared CD40 ARC samples from two mouse B cell lines, one stably expressing a hybrid CD40 molecule composed of the extracellular domain of human CD40 fused to the transmembrane and cytoplasmic domains of mouse CD40 (hmCD40)[8], and a second line expressing a hybrid lacking all but 7 amino acids of the 74 amino acid cytoplasmic domain (hmCD40Δ67; a similarly truncated human CD40 molecule has no detectable signaling activity [9]). Proteins captured with full-length hmCD40 and not the truncated molecule potentially contribute to CD40 signaling. The human extracellular domain of the hybrid molecules allowed ARC purification with anti-human CD40 antibody, precluding co-purification of the endogenous mouse CD40 expressed by the cell lines.

**Figure 1:**
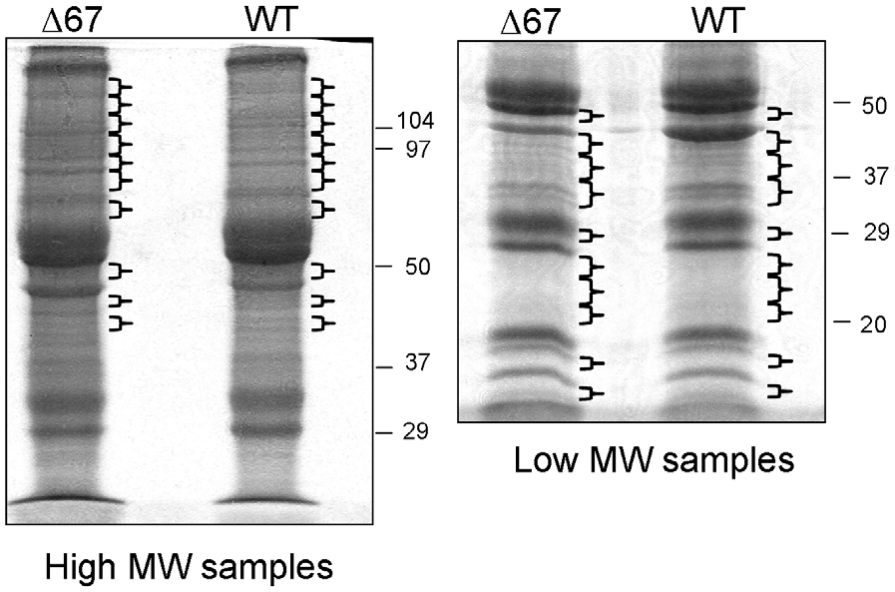
SDS-PAGE fractionation of CD40 ARC samples for analysis by mass spectrometry. Lanes containing anti-hCD40 ARC samples from CH12.LX cells transfected with hmCD40 (WT) or hmCD40Δ67 cells (Δ67) are indicated. Brackets indicate approximate positions of gel slices analyzed. High molecular weight proteins were analyzed in the first experiment (left panel), while lower molecular weight material was analyzed in a second experiment (right panel).

**Figure 2:**
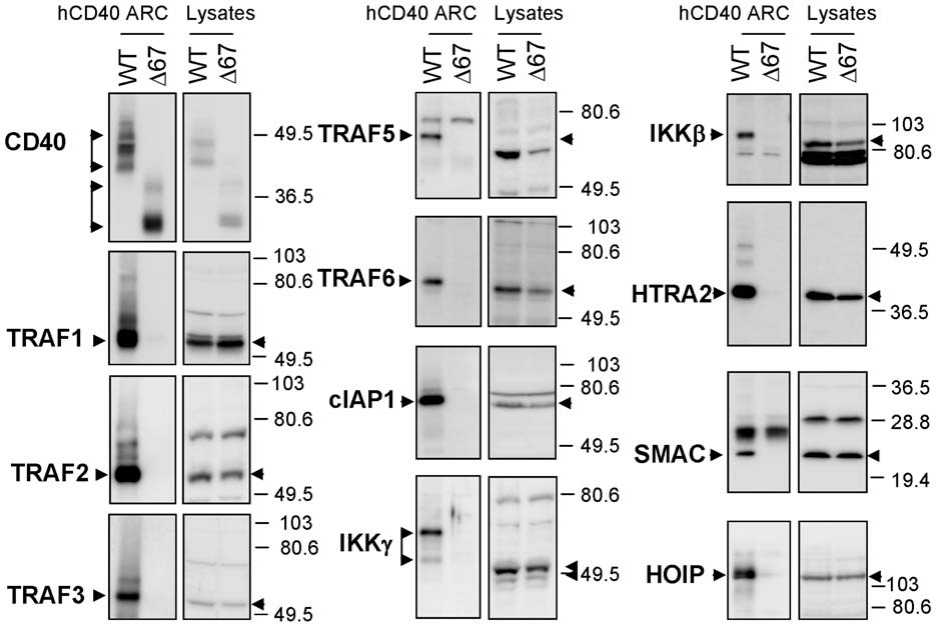
Western blot verification of mass spectrometry results. hmCD40 and hmCD40Δ67 ARC samples (3.3×10^6^ cell equivalents per lane) were fractionated by SDS-PAGE and transferred to PVDF membrane for Western blotting with the indicated antibodies. Lanes containing whole cell lysates (1.0×10^5^ cell equivalents per lane) were run in parallel. Each blot is representative of three or more experiments.

ARC-purified hmCD40 and hmCD40Δ67 samples were fractionated by SDS-PAGE and processed for liquid chromatography/tandem mass spectrometry (Fig. 1). We analyzed material from two independent ARC purifications. In the first, material >40 kD was examined. In the second, we analyzed lower molecular weight (∼15–50 kD) material. Under the low-stringency conditions used, the majority of the proteins associated with full-length and truncated CD40 appeared similar on gels stained for total protein. However, we hypothesized that mass spectrometry would be useful in revealing subtle but important differences between the proteins associated with full-length and truncated CD40. In collecting gel slices for analysis, we attempted to exclude portions of the gel where pronounced protein bands were detected in both the control and full-length CD40 lanes, thereby limiting the peptide signals from high-concentration proteins. Peptide signatures detected in the material associated with full-length CD40 but not truncated CD40 are listed in Table 1. Mass spectrometry and Western blot analysis of ARC-purified CD40 complexes both showed that known CD40-binding proteins, including TRAFs 1, 2, 3, 5, 6, and cIAP1, co-immunoprecipitated with full length hmCD40 but not with hmCD40Δ67, validating the purification method (Fig. 2). Some of the ARC-purified proteins, including the TRAFs, appeared as one or more higher molecular weight forms (on Western blots) in comparison to the corresponding proteins in whole-cell lysates from unstimulated cells. Several of these proteins are known to undergo activation-induced post-translational modifications, including ubiquitination [10], potentially accounting for the molecular weight shifts. Our mass spectrometry analysis also revealed proteins not previously shown to interact with CD40, including SMAC, HTRA2 and HOIP. We verified these targets by Western blot analysis of material recovered by ARC, which showed that each of the three proteins associated with full-length CD40 but not the truncated form (Fig. 2). Thus, both mass spectrometry and Western blot analysis of CD40 complexes recovered by ARC indicate that SMAC, HTRA2 and HOIP interact with the cytoplasmic tail of CD40 upon receptor aggregation.

**Table 1:** Proteins uniquely associated with ARC-purified hmCD40 as determined by mass spectrometry analysis.

Protein Name*	NCBI Accession	MW (Da)	Unique peptides
HTRA2	GI:20141609	49330.8	25
RNF31/PAUL/HOIP	GI:14017768	118130.5	17
cIAP1/BIRC2	GI:160333366	69659	15
TRAF5	GI:30580619	64126.3	13
TRAF3	GI:114842405	64276.1	9
IKK*β*	GI:116283233	81038.4	6
TRAF2	GI:26332439	56008	5
CD40	GI:13016736	28875.9	4
SMAC/DIABLO	GI:85677504	26802.3	4
TRAF6	GI:30580620	60064.6	3
IKKα	GI:26330608	84755.9	3
TRAF1**	GI:148676689	45786.5	13

*All protein assignments listed met or exceeded the Scaffold 95% confidence filter.

**TRAF1 was detected in gel slices containing material at or above the known molecular weight for TRAF1 in hmCD40 ARC samples, but was also detected in both hmCD40Δ67 and hmCD40 gel slices containing material of approximately 35 kD.

### TRAF2 is important for the association of novel proteins with CD40

**Figure 3:**
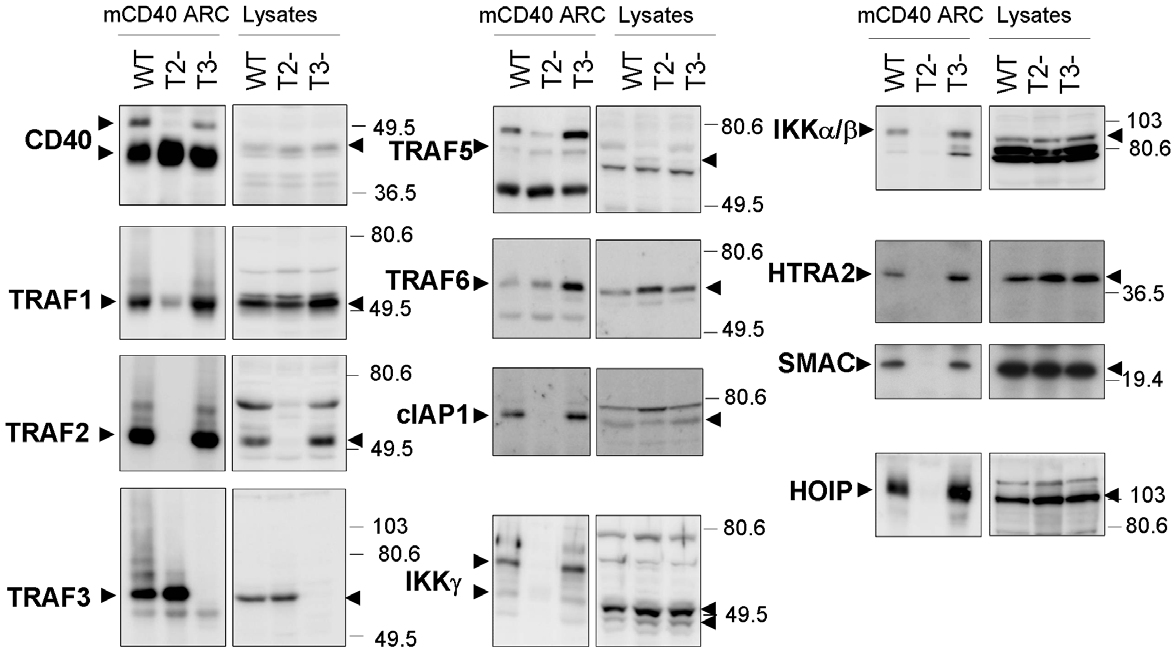
TRAF-dependent recruitment of proteins to CD40. Endogenous CD40 from CH12.LX cells (WT), TRAF2-deficient CH12.LX (T2-), or TRAF3 deficient CH12.LX cells (T3-) was isolated using ARC (lane loading as per Fig. 2). Western blotting was performed with the indicated antibodies. Each blot is representative of three or more experiments.

Proteins associated with the CD40 signaling complex could bind directly to CD40 or may be recruited through other proteins such as the TRAFs. Using TRAF2- and TRAF3-deficient B cell lines [8], [11] and CD40 ARC, we found that TRAF2 participates in the recruitment of HOIP, HTRA2, SMAC, TRAF1, cIAP1, and two IKK proteins (Fig. 3). The decreased recruitment of HTRA2 and SMAC to CD40 in TRAF2-deficient B cells may be related to the decreased recruitment of cIAP1, a potential binding partner for both HTRA2 and SMAC [12], [13], [14].

The recruitment of TRAF3 to CD40 was also altered in TRAF2-deficient B cells. In cells expressing TRAF2, much of the TRAF3 associated with CD40 appeared to be post-translationally modified, as evidenced by high molecular weight forms on Western blots. Ubiquitination of TRAF3 may account for this high molecular weight material [15]. In contrast, the TRAF3 associated with CD40 in TRAF2-deficient cells appeared largely unmodified, suggesting that TRAF2, perhaps through associated factors including SMAC, HTRA2 and HOIP, influences the function of TRAF3.

In contrast to the results obtained from TRAF2-deficient cells, analysis of CD40 complexes obtained by ARC from TRAF3-deficient B cells revealed that TRAF3 was not responsible for the recruitment of any of the other proteins we assayed, which included SMAC, HTRA2 and HOIP. However, in the absence of TRAF3, the amount of CD40-associated TRAF6 appeared to increase. This observation is consistent with the previously identified role of TRAF3 as a negative regulator of CD40-mediated B cell activation [2].

### HOIP facilitates CD40-mediated NF-κB activation

**Figure 4:**
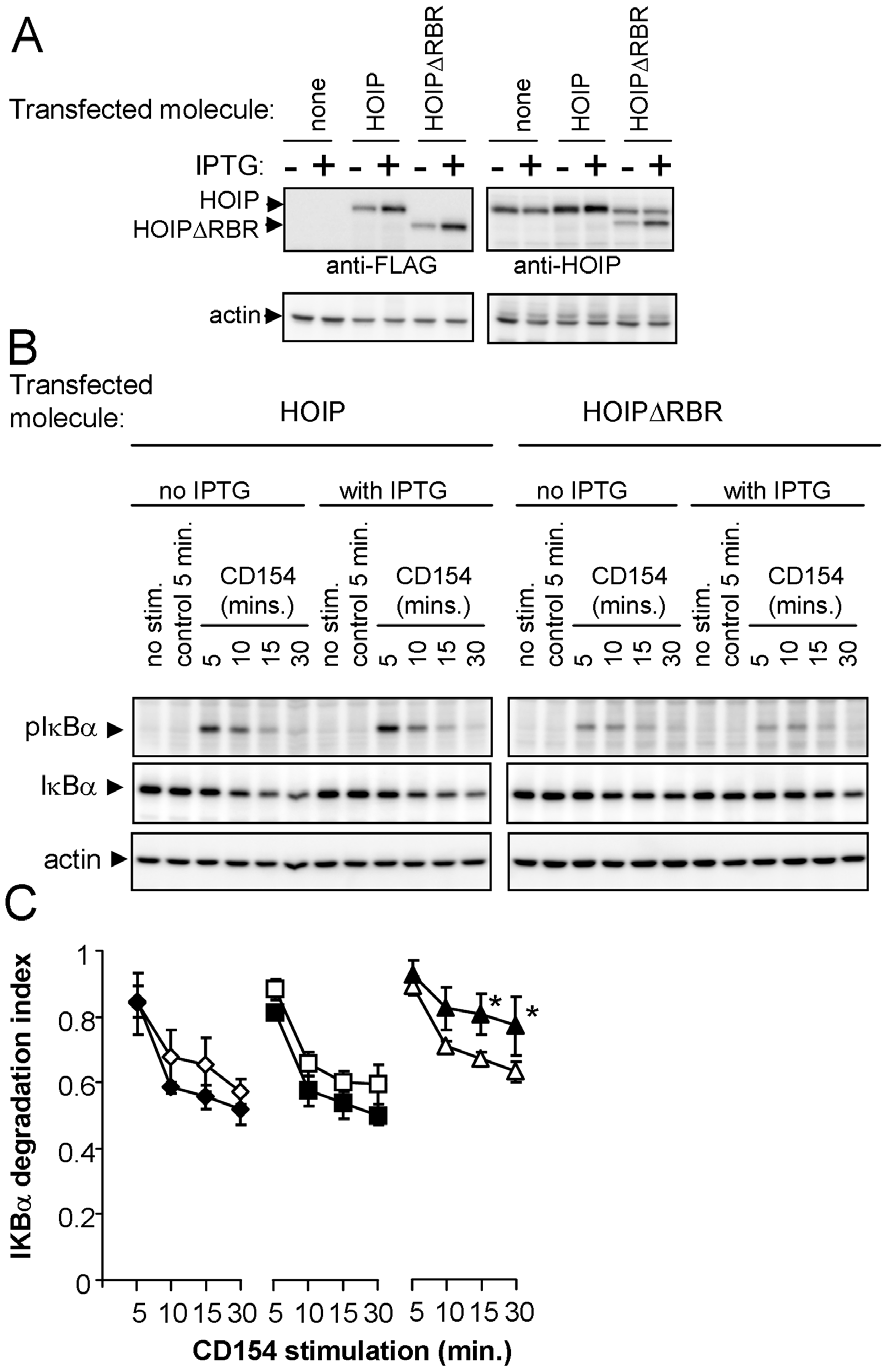
HOIPΔRBR inhibits IκBα phosphorylation and degradation. TRAF6-deficient A20.2J cells were stably transfected with FLAG2X-tagged HOIP or HOIPΔRBR in an IPTG-inducible expression vector. A, Expression of FLAG2X-tagged HOIP or HOIPΔRBR in stably transfected cell lines. Western blots of cell lysates from uninduced and IPTG-induced cell lines were probed with anti-FLAG (left) and anti-HOIP (right). Blots were reprobed for actin to verify equivalent lane loading. B, The two cell lines were stimulated with CD154 (CD40 ligand) for the times indicated and then processed for Western blotting. Unstimulated cells (first lane) and cells incubated for 5 minutes with insect cells lacking CD154 served as negative controls. Western blotting for phosphorylated IκBα was performed; blots were then stripped and reprobed for total IκBα and actin. C, Quantification of IκBα degradation (panel B) in TRAF6-deficient A20.2J cells stably transfected with Lac repressor only (◊;♦), Lac repressor plus full-length HOIP (□;▪), or Lac repressor plus HOIPΔRBR (Δ; ▴). Filled symbols indicate cultures pre-treated with IPTG. IκBα bands in each lane were normalized to the actin signal. The degradation index is the fraction of IκBα remaining at each time point relative to the amount of IκBα present in cells incubated for 5 minutes with insect cells lacking CD154. The results presented are the mean of four experiments. Error bars indicate standard error of the mean.*, p<0.05 (one-sided Student's t test).

One of the molecules we found associated with CD40 is the putative ubiquitin ligase HOIP. Recently, a protein complex containing HOIP and the ubiquitin ligase HOIL was shown to activate the IKK complex through a ubiquitin-dependent mechanism [16]. Interestingly, the HOIP-containing complex mediates the assembly of a unique form of polyubiquitin in which individual ubiquitin molecules are linked in a head-to-tail fashion [17]. To test if HOIP participates in the CD40-mediated activation of NF-κB, we stably transfected a mouse B cell line with an inducible HOIP cDNA construct or a similar HOIP construct lacking most of the ‘ring between ring fingers’ (RBR) domain necessary for ubiquitin ligase activity [16], [17]. Previous work showed that CD40 can activate NF-κB through either TRAF2 or TRAF6 [4]. To specifically examine the TRAF2-dependent pathway, the IPTG-inducible HOIP expression constructs were stably transfected into TRAF6-deficient A20.2J cells [4] (Fig. 4a). CD40-mediated activation of NF-κB, as measured by phosphorylation and degradation of IκBα, was unchanged in the cell line expressing wild-type HOIP, and diminished in cells expressing the HOIPΔRBR mutant (Fig. 4b, c). The reduction in NF-κB activation by HOIPΔRBR was not due to clonal variation, as IPTG induction of HOIPΔRBR expression significantly reduced IκBα degradation over that observed in the absence of IPTG. Although the inhibition of CD40 signaling by HOIPΔRBR was modest, the level of inhibition was consistent with the modest level of IPTG-induced HOIPΔRBR expression (slightly greater than that of endogenous HOIP; Fig. 4a).

The inhibition of CD40-mediated NF-κB activation by HOIPΔRBR suggests that the ubiquitin ligase activity of HOIP has a role in the CD40-mediated activation of NF-κB. In support of this hypothesis, we cataloged 132 mass spectra matching ubiquitin (each spectrum with a peptide identification probability of 95% or greater as determined by Scaffold) in the hmCD40 ARC samples (high MW gel slices, Fig. 1, left panel), while only 4 spectra from the hmCD40Δ67 control matched ubiquitin. Furthermore, a peptide signature consistent with linear polyubiquitination was detected in the hmCD40 sample (two spectra matching the peptide GGMQIFVK with approximately 80% confidence). Although further work will be necessary to confirm the presence of linear polyubiquitin in the CD40 signaling complex, the two matching spectra suggest that CD40 may employ this novel form of polyubiquitination in the activation of signaling.

## Discussion

The first CD40-associated signaling protein (TRAF3) was reported in 1994 [1]. Since that time, additional CD40-interacting proteins have been identified, including TRAFs 1, 2, 5, 6, cIAP1, and subunits of the IKK complex. Using our ARC immunoprecipitation method, we have identified three additional proteins that may be involved in CD40 signaling. These new interactions can now be further evaluated in additional cell line models and in primary cells. Two of the CD40-associated proteins we identified, HTRA2 and SMAC, may have roles in the regulation of apoptosis by CD40. HTRA2 is a putative serine protease and can bind cIAP1 [13], which may be the means by which it is recruited to CD40. Similarly, SMAC is a known regulator of apoptosis and also binds cIAP1 under some conditions [12], [14]. The roles of cIAP1, HTRA2, and SMAC in CD40 signaling remain to be fully explored. However, CD40 signaling has been shown to both enhance [18] and inhibit [19] cellular apoptosis. Potentially, cIAP1 contributes to the protection from apoptosis afforded by CD40 signals, while SMAC and HTRA2 counteract this activity [20], [21].

We also found that the ubiquitin ligase HOIP associates with CD40. HOIP RNA is broadly expressed in a variety of tissues [22] but relatively little is known about the physiologic function of the protein. Interestingly, HOIP (or a splice variant) may be overexpressed in certain types of cancer [23], suggesting a role in cellular activation or cell proliferation. HOIP may also regulate the activity of the transcriptional repressor DAX-1 [22], and may therefore have a role in reproductive development and steroidogenesis. Recently, HOIP was shown to be recruited to TNFR1 in TNF-stimulated cells [24], and to be part of an enzyme complex capable of producing linear polyubiquitin chains which can participate in the activation of the IKK complex [16], [17]. We did not detect the other major component of the linear polyubiquitination complex, a protein known as heme-oxidized IRP2 ubiquitin ligase-1 (HOIL-1), in our mass spectrometry samples. Either HOIP is recruited to CD40 in the absence of HOIL, or technical issues prevented detection of HOIL. We are currently addressing these possibilities. Nevertheless, our experiments with HOIPΔRBR suggest that the ubiquitin ligase activity of HOIP contributes to the CD40-mediated activation of NF-κB.

Our results with TRAF2-deficient cells indicate that TRAF2 is critical for the recruitment of several proteins to the cytoplasmic domain of CD40, including HOIP. TRAF2 does not appear to be constitutively associated with CD40, but is recruited upon engagement of CD40 by ligand or agonistic antibody [6]. Therefore, it follows that the interaction of HOIP with CD40 is largely (if not entirely) dependent upon receptor activation. As previously shown, CD40 is capable of recruiting IKKβ and IKKγ and their recruitment is similarly TRAF2-dependent [3]. If the recruitment of the IKK proteins together with HOIP facilitates the activation of NF-κB, one might expect that CD40-mediated NF-κB activation would be defective in TRAF2-deficient B cell lines. This is not the case [8]. However, both TRAF2 and TRAF6 can mediate CD40-stimulated NF-κB activation, and the physical binding of TRAF6 to CD40 appears unnecessary for this activity [4]. Potentially, the TRAF2-mediated activation of NF-κB involves recruitment of IKK subunits to CD40, while TRAF6-mediated activation of NF-κB does not. We are currently working to define the mechanism by which CD40 signals through TRAF6 in the absence of a direct interaction between the two proteins, and to determine if HOIP is required for the activation of NF-κB via this pathway.

## Materials and Methods

### Cell lines

The mouse B cell lines A20.2J, CH12.LX, TRAF2-deficient CH12.LX, TRAF3-deficient CH12.LX, and TRAF6-deficient A20.2J stably expressing the bacterial Lac repressor have been described [4], [8], [9], [11], [25].

### DNA constructs and transfections

An expression plasmid encoding hmCD40 has been described [8]. PCR mutagenesis was used to create a similar construct in which 67 amino acids were deleted from the cytoplasmic domain. CH12.LX cells were stably transfected with hmCD40 expression vectors as described [4], [8]. An expression vector encoding mouse HOIP with an amino terminal 2X FLAG tag was prepared by PCR-mediated mutagenesis. A mutant HOIP construct (HOIPΔRBR) similar to a mutant lacking ubiquitin ligase activity [16], [17] was prepared by removing a SalI fragment from full-length HOIP cDNA, resulting in the deletion of amino acids 688-888. HOIP cDNA constructs were ligated into pOPRSV5.neo, an IPTG-inducible expression vector [4]. Expression of IPTG-inducible constructs was initiated by overnight incubation of cells with 100 µM IPTG. TRAF6-deficient A20.2J cells expressing Lac repressor were stably transfected with the HOIP constructs as described [4], [8].

### Activated receptor capture (ARC)

Protein G magnetic beads (Dynal) were coated with anti-hCD40 mAb (G28-5 (ATCC); 7.5–10 µg/10 µl beads), or goat-anti-rat IgG (Jackson ImmunoResearch) followed by rat anti-mouse CD40 mAb (1C10; [26]) as per manufacturer's protocols (immunoprecipitation by 1C10 is most efficient when beads are pre-coated with an anti-rat IgG (data not shown)). Cells expressing hmCD40 molecules were incubated in culture medium with anti-hCD40 Ab-coated beads for 60 minutes at room temperature. Following incubation, beads and cells were pelleted by centrifugation (2 min. at 300×g) and the medium discarded. Cells were lysed and beads washed as described [4]. Bead-bound proteins were eluted in SDS-PAGE sample loading buffer and heat denatured. Immunoprecipitates were fractionated by SDS-PAGE. For proteomic analysis, gels were stained with GelCode Blue (Pierce). Gel slices (∼1 mm×1 mm×5 mm) were cut from the lanes as shown in Fig. 1 and then processed for mass spectrometry by the Michigan State University Proteomics Facility (East Lansing, MI). For proteomic samples, 1.8×10^8^ cells were stimulated in 2.0 ml culture medium with 150 µl anti-hCD40 beads (100 µg Ab). Cells were lysed in 2 ml lysis buffer. For Western blot analysis, 1-5×10^7^ cells were stimulated with anti-CD40 beads (10 µl per 10^7^ cells) in 0.4 ml culture medium; cells were lysed and beads were washed as above with 0.4 ml volumes of lysis buffer.

### NF-κB assays

Cells were stimulated with mCD154 (CD40 ligand) expressed on HI-5 insect cells as previously described [4], [8]. Briefly, 5×10^4^ HI-5 insect cells or HI-5-mCD154 cells were added to 1×10^6^ B cells in 1 ml culture medium and pelleted by centrifugation for 1 minute at 300×g. The cells were incubated at 37°C for the indicated times, and lysed in 100 µl 2X SDS-PAGE sample buffer. Lysates were sonicated to shear DNA and heated for 5 min at 95°C. Lysates were fractionated by SDS-PAGE and transferred to polyvinylidine fluoride (PVDF) membranes for Western blotting.

### Western blots

Western blotting was performed as previously described [6]. Antibodies specific for TRAF2 and TRAF6 were from Medical and Biological Laboratories, Ltd. Antibodies to TRAF3 (H-122), TRAF1 (N-19), IKKγ (FL-419), IKKα/β (H-470), and CD40 (H-120), were from Santa Cruz Biotechnology. Antibodies to HTRA2, cIAP1, SMAC/DIABLO, and actin were from Epitomics, Proteintech Group, BD Biosciences, and Chemicon International, respectively. Antibodies to ALIX and IκBα were from Cell Signaling. A pGEX-6P1 vector encoding mouse HOIP(Met^1^-Ser^333^) preceded by –Glu-Phe- and followed by –Ala-Ala-Ala was used to produce a GST fusion protein; following purification, the fusion protein was cleaved with PreScission Protease (GE Healthcare Life Sciences). HOIP antiserum was isolated from rabbits immunized with the N-terminal fragment of HOIP at Covance Research Products, Inc. HRP-labeled antibodies were from Jackson ImmunoResearch. Chemiluminescent HRP substrate was from Pierce. Images of Western blots were collected with an LAS-4000 low-light camera system and analyzed with Multigauge software (FujiFilm Medical Systems).

### Proteomic analysis

Gel bands as shown in Fig. 1 were subjected to in-gel tryptic digestion [27]. The extracted peptides were injected into a MICHROM Paradigm Platinum Peptide Nanotrap (C18, 5Å, 0.15×50 mm) and washed for 5 minutes with 85% water (buffer A), 5% acetonitrile (buffer B), 10% buffer C (1% formic acid) at 20 µl/min. The bound peptides were eluted onto a 10 cm×75 µm picofrit column (New Objectives) packed with Microm Magic C18 AQ and eluted over 30 minutes with a gradient of 5% to 90% buffer B, with constant 10% buffer C in 22 minutes using a Michrom Paradigm MDLC (300 nL/min flow rate). Eluted peptides were sprayed into a LTQ linear ion trap mass spectrometer (ThermoFisher) with a nanospray source. The top five ions in each survey scan were subjected to data-dependant zoom scans followed by low energy collision induced dissociation. The resulting MS/MS spectra were converted to peak lists using BioWorks Browser v 3.2 (ThermoFisher) and the default LTQ instrument parameters. Peak lists were searched against all mouse protein sequences available from NCBInr (downloaded 11/14/2008) using the Mascot searching algorithm, v2.2 (Matrix Science, Inc.). Search parameters allowed up to 2 missed tryptic cleavages; variable oxidation and carbamidomethylation of methionine and cysteine, respectively; peptide tolerance of +/− 200 ppm; MS/MS tolerance of 0.8 Da; peptide charge state limited to +2/+3. The Mascot output was analyzed using Scaffold (Proteome Software, Inc.) to probabilistically validate protein identifications using the ProteinProphet [28] algorithm. Proteins with assignments above the Scaffold 95% confidence filter were considered present in the sample.
